# Hydrofluoric acid burns in the western Zhejiang Province of China: a 10-year epidemiological study

**DOI:** 10.1186/s12995-016-0144-3

**Published:** 2016-12-07

**Authors:** Yuanhai Zhang, Jianfen Zhang, Xinhua Jiang, Liangfang Ni, Chunjiang Ye, Chunmao Han, Komal Sharma, Xingang Wang

**Affiliations:** 1 Department of Burns & Plastic Surgery, Zhejiang Quhua Hospital, Quzhou, 324004 China; 2 Department of Burns & Wound Care Center, Second Affiliated Hospital of Zhejiang University College of Medicine, Hangzhou, 310009 China; 3 Zhejiang University School of Medicine, Hangzhou, 310000 China

## Abstract

**Background:**

Chemical burns caused by hydrofluoric acid (HF) frequently occur in the Western Zhejiang Province. This study aimed to investigate the epidemiological characteristics of HF burns within this region.

**Methods:**

A 10-year retrospective analysis was conducted using data from all inpatients with HF burns. These patients were treated at the Department of Burns and Plastic Surgery at our hospital between January 2004 and December 2013. Information obtained for each patient included sex, age, occupation, burn location, burn cause, and the hazard category of the chemical which caused the burn. Data regarding wound site and size, accompanying injuries, serum electrolyte levels, operations, length of hospital stay, and mortality were also assessed.

**Results:**

A total of 201 patients (189 males, 12 females; average age: 38.33 ± 10.57 years) were admitted due to HF burns. Over the 10-year period, the morbidity of HF burns in the past 10 years showed a gradual increase, which paralleled the development of local fluoride industries. Most HF injuries were work related and distributed in working-age patients. Aqueous HF solutions, especially highly concentrated ones, were the most common chemical cause of HF burns. Moreover, inappropriate operation, machine problems, and inadequate protection were identified as the leading causes of HF burns in the workplace. The burn area was <5% of TBSA in more than 90% of patients, and the most common burn sites were the head, neck, and upper extremities. Approximately 17% of patients underwent surgical operation. Accompanying injuries should be detected and treated correctly in a timely manner. Lastly, electrolyte imbalances, such as hypocalcaemia, hypomagnesaemia, and hypokalaemia, occurred frequently in patients with HF exposure; however, hyperkalaemia was not encountered in this study.

**Conclusion:**

Based on the epidemiological results for HF burns in this region, the related enterprises and local authorities should be encouraged to upgrade management policies and to provide necessary occupational hazard education and safety training for high-risk occupations within high-risk working populations. Furthermore, the enhancement of hazardous chemicals management is also needed. Finally, for patients with HF exposure, early and correct pre-hospital triage, treatment and consequent in-hospital treatment and procedures should also be improved.

## Background

Hydrofluoric acid (HF), an important industrial material, is used widely in chemical industries including: electronics manufacturing, glass etching, smelting, cleaning, and other industrial fields [1–3]. HF is very dangerous chemical, not only causing local tissue corrosion, but also systemic poisoning by ongoing absorption into the human body. Previous studies have shown that HF burns over small areas can even result in death [4, 5]. In some regions throughout the world, HF has been listed as the top cause of chemical burns [6, 7]. Our previous research investigated the epidemiological features of chemical burns occurring in Zhejiang Province between September 2008 and August 2009, where results showed that HF was the leading cause of chemical burns (27.4%, 135/492 patients) within the research population [8]. Thus, the hazard behind direct exposure to HF can be contributed towards the industrial structure of this region.

Zhejiang Province, located in south-eastern China, is famous for its thriving chemical industries. By using its rich resources of fluorite, HF can be produced when reacted with concentrated sulphuric acid [9]. This region in China has therefore become the largest industrial producer of fluoride, with an annual HF output of 400,000 tons [10]. With this rapid development of fluoride industries and high turnover of chemical usage within the Zhejiang region, the incidence of chemical burns has increased dramatically. Events with severe casualties caused by HF have been reported [11, 12].

To clarify the epidemiological characteristics of chemical burns in western Zhejiang Province, a 10-year retrospective analysis was conducted. The analysis included data from patients with chemical burns admitted to our hospital’s department of Burns and Plastic Surgery between January 2004 and December 2013. Preliminary results showed that the morbidity rate of chemical burns was as high as 18.6% (690 cases), and HF remained the leading cause of chemical injuries (201 cases, 29.13%) in this period and amongst the cases that were seen [10]. In this study, we further analysed the epidemiological features of the 201 inpatients with HF burns, with the aim of providing further evidence to support the upgrade of safety measurements and the formulation of preventive strategies within the Zhejiang Province.

## Methods

A 10-year retrospective analysis including all patients with HF burns admitted to the Department of Burns and Plastic Surgery between January 2004 and December 2013 was conducted. Information obtained for each patient included sex, age, occupation, location of burn, cause of burn, and the category of chemical that caused the burn. Data regarding wound site and size, accompanying injuries, serum electrolyte levels, operations, length of hospital stay, and mortality were also assessed. All of the data mentioned above were collected and rechecked by two medical staff. The data recorded in the Excel form were further analysed by the statistician.

## Results

### Trends of HF burns

**Fig. 1:**
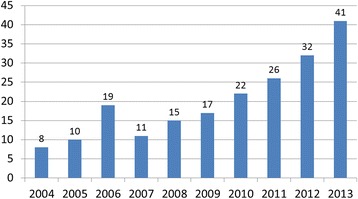
The number of hydrofluoric acid burns from January 2004 to December 2013

Figure 1 shows the number of patients admitted each year due to HF burns. It can be seen that the incidence of cases increased gradually over the 10-year period, although a slight fluctuation in this trend was observed in 2006. The overall number of patients with HF exposure has however, increased rapidly, especially in the recent years.

### Age, sex, and education

Among the 201 patients, 189 were male and 12 were female (ratios of 15.75:1). The average age was 38.33 ± 10.57 years, with a range of 1.5–69 years. HF burns occurred most frequently in patients aged 30–39 years (33.83%), followed by those aged 40–49 years (32.84%), 20–29 years (17.41%), and 50–59 years (11.44%; Table 1).

**Table 1:** The age distribution of patients with HF burns

Age (Years)	Csaes	
	N	Percent (%)
<10	2	0.99
10 ~ 19	4	1.99
20 ~ 29	35	17.41
30 ~ 39	68	33.83
40 ~ 49	66	32.83
50 ~ 59	23	11.44
>60	3	1.49

**Fig. 2:**
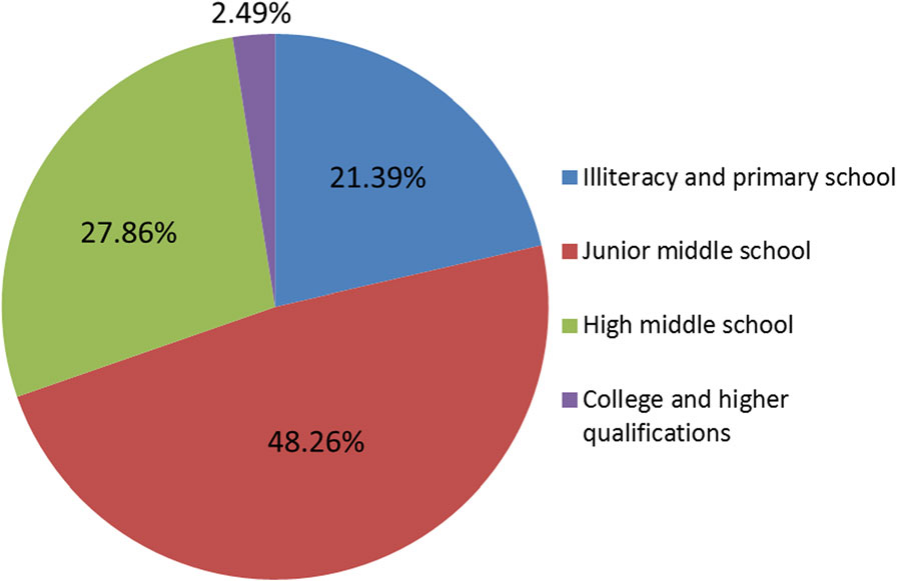
Educational background of patients with hydrofluoric acid burns

Figure 2 shows the education levels of these patients. The majority of patients had low levels of education (illiteracy/primary school education, 21.39%; junior middle school education, 48.26%), 27.86% of patients had completed middle school / high school, and only 2.49% had tertiary education of college/university degrees or higher.

### Sources of patients and causes of HF burns

Out of all the cases presented in our department, the most frequent source of HF burns was seen as work related (Table 2). More than half (109 cases, 54.23%) of these patients worked in the fluorine industry; followed by stevedoring and transportation industries (35 cases, 17.41%); the semiconductor industry (18 cases, 8.96%); metal rust removal industries (13 cases, 6.47%); glass etching industries (12 cases, 5.97%), and finally followed by waste and disposal service sectors (8 cases, 3.98%), and other sectors and industries (6 cases, 2.98%).

**Table 2:** Sources of patients with HF burns

Sources	Cases	
	N	Percent (%)
Fluorine industry	109	54.23
Stevedoring and transportation	35	17.41
Semiconductor industry	18	8.96
Metal rust removing	13	6.47
Glass etching	12	5.97
Waste and disposable service	8	3.98
Others	6	2.98

**Fig. 3:**
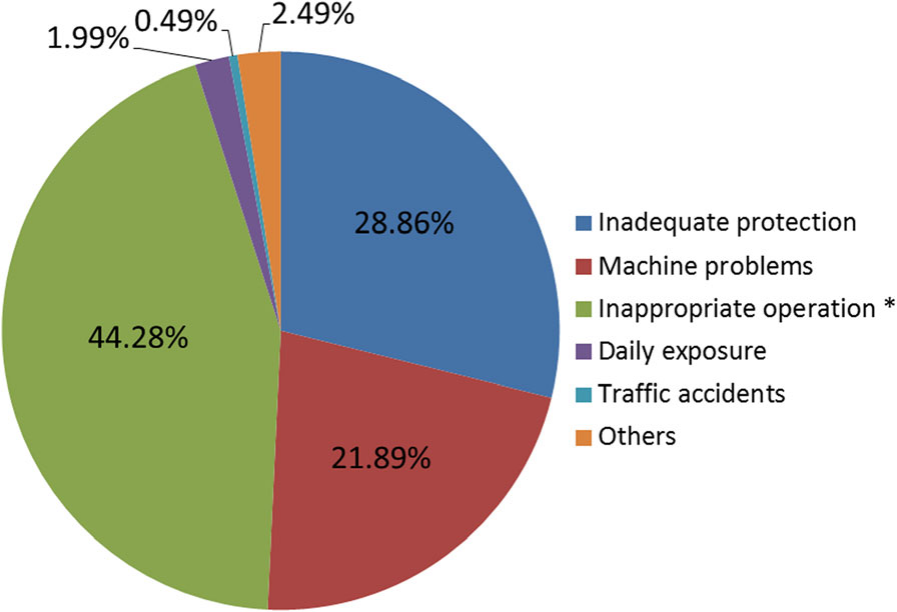
Causes of hydrofluoric acid burns

Furthermore, more than 95% of HF burns in this study were due to three major causes: (i) inappropriate operation (89 cases, 44.28%), (ii) inadequate protection (58 cases, 28.86%), and (iii) machine problems (44 cases, 21.89%). Less than 5% of cases were caused by daily exposure, traffic accidents, or other reasons (Fig. 3).

Table 3 presents the categories of chemicals that caused HF burns. The majority (177 cases, 88.06%) of HF injuries were caused by aqueous HF solutions, followed by mixtures containing HF (18 cases, 8.96%). Chemical burns caused by hydrogen fluoride accounted for the least (2.98%) of these injuries.

**Table 3:** Categories of chemicals causing HF burns

Chemicals	Cases	
	N	Percent (%)
Hydrogen fluoride	6	2.98
HF solution	177	88.06
Mixture		
HF+ sulphuric acid (H_2_SO_4_)	10	4.98
HF+ hydrochloric acid (HCl)	2	0.99
HF + dimethylamine	1	0.49
HF + Trichloroethylene	1	0.49
HF + pentafluoropropanol	1	0.49
HF+ unknown chemicals	3	1.49

The concentrations of HF in the various solutions were analysed further; where the results are presented in Table 4. Aside from the 72 cases caused by unknown concentrations, high-concentration (>50%) solutions caused the majority (66 cases, 32.84%) of HF burns, followed by solutions with moderate (41 cases, 20.4%) and low (16 cases, 7.96%) concentrations of HF.

**Table 4:** Distribution of HF concentration

HF concentration (%)	Cases	
	N	Percent (%)
<20	16	7.96
20 ~ 50	41	20.40
>50	66	32.84
Unknown	72	35.82
Hydrogen fluoride	6	2.98

### Sites and extent of HF burns

**Fig. 4:**
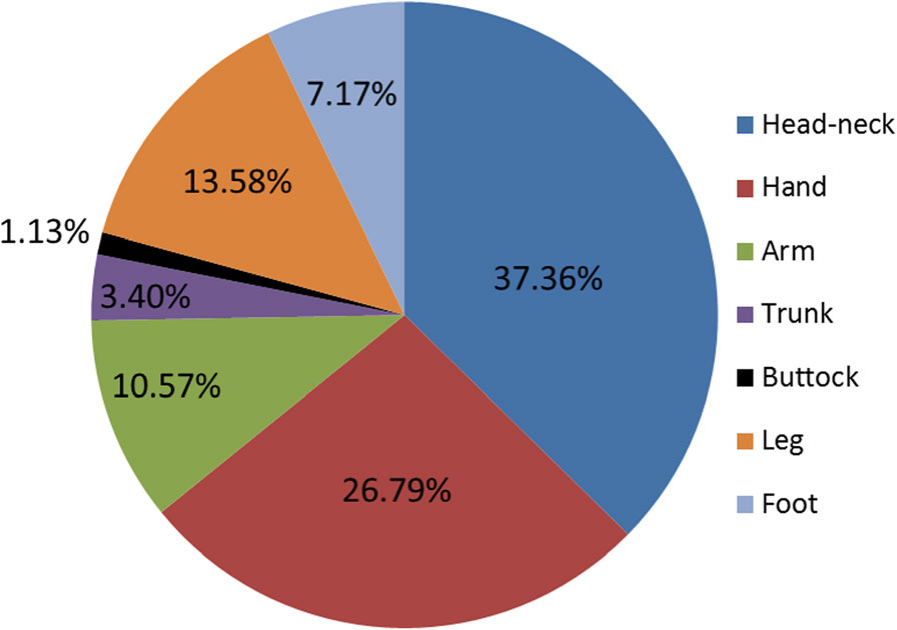
Distribution of burn sites in patients with hydrofluoric acid burns

HF burns were observed at a total of 265 sites among the 201 patients (Fig. 2). The most common sites of injury were the head and neck (99 cases, 37.36%), hands (71 cases, 26.79%), legs (36 cases, 13.59%), arms (28 cases, 10.57%), foot (19 cases, 7.17%, trunk (9 cases, 3.40%), and buttocks (3 cases, 1.13%); Fig. 4).

Burn areas were calculated by estimating the sizes of first, second, and third degree burns. Table 5 shows the burn area distribution for these patients. In all patients, burns covered <1 to 42% of the total body surface area (TBSA). One hundred and twenty-one (60.20%) patients had burns covering <1% of the TBSA, and 61 (30.35%) patients had burns covering 1–5% of the TBSA. Only 19 (9.45%) cases had burn areas of >5% TBSA.

**Table 5:** TBSA distribution for patients with HF burns

TBSA^a^ (%)	Cases	
	N	Percent (%)
<1	121	60.2
1–5	61	30.35
6–10	9	4.48
11–20	4	1.99
21–30	3	1.49
31–40	1	0.49
>40	2	0.99

^a^Calculation of TBSA refers to all the burned area involved, including the first, second and third degrees

### Accompanying injuries

Injuries accompanying HF burns included ocular injuries (17 cases, 8.46%), inhalation injuries (9 cases, 4.48%), and digestive tract injuries (1 case, 0.50%).

### Seasonal distribution

**Fig. 5:**
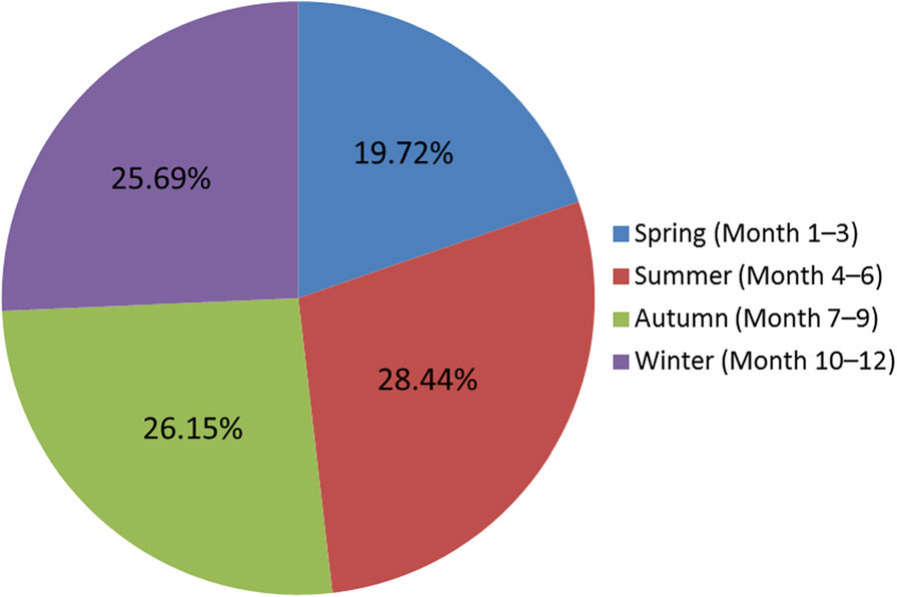
The seasonal distribution of hydrofluoric acid burns

HF burns occurred more frequently in the summer (28.44%), autumn (26.15%), and winter seasons (25.69%) than in the spring season (19.72%; Fig. 5).

### Pre-hospital treatment

**Fig. 6:**
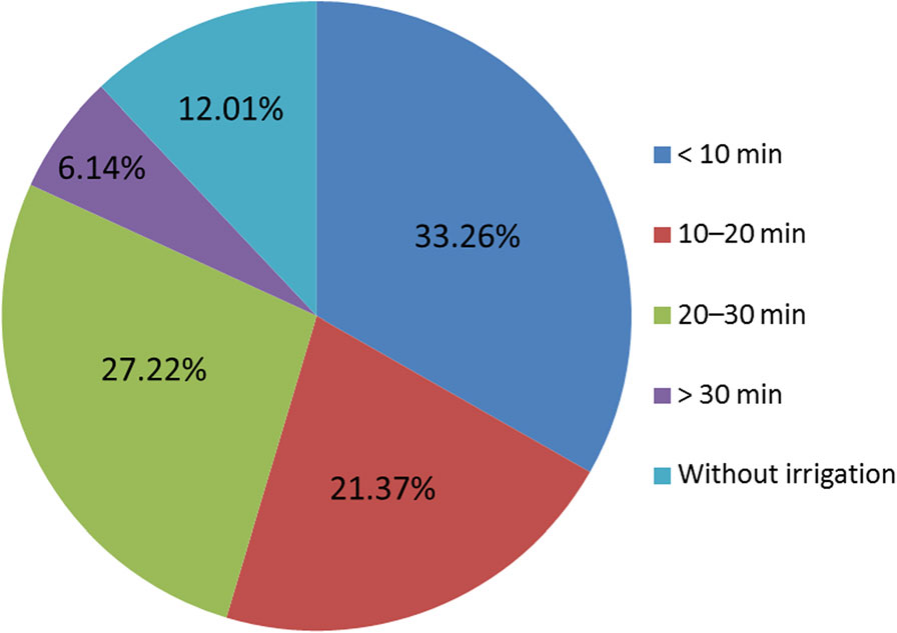
The prehospital water irrigation for hydrofluoric acid burns

In nearly 88% of cases, on-site water irrigation was immediately performed after HF burns had occurred. Amongst these cases, there were 8 patients who received immediate washing of their chemical burns by health professionals: First with Hexafluorine^®^ solution, followed by tap water. However, for all of the patients who received water irrigation treatment, the rinsing time varied greatly (Fig. 6).

### Serum electrolyte levels

Results of electrolyte tests conducted within 3 days after exposure were analysed. The morbidity rates of hypocalcaemia, hypomagnesaemia, hypokalaemia, and hyperkalaemia were calculated and are listed in Table 6. Hypocalcaemia (47 cases, 23.38%) and hypomagnesaemia (28 cases, 13.93%) occurred more frequently in patients with HF burns. Only 18 (8.96%) patients had hypokalaemia, and no patient had hyperkalaemia.

**Table 6:** The situations of serum electrolytes for patients with HF burns (*n* = 201)

Electroyte imbalance	Cases	
	N	Percent (%)
Hypocalcemia	47	23.38
Hypomagnesemia	28	13.93
Hypokalemia	18	8.96
Hyperkalemia	0	0

### Number of operations

Thirty-five (17.41%) patients underwent surgical operations. Among these, 9 cases were treated with urgent escharotomy and skin grafting. 7 cases were treated with flap transfers for wound reconstruction, and 11 cases were treated with split-thickness skin grafting for wound closure.

### Length of hospital stay

The average hospital stay was 8.9 ± 10.7 days, with a range of 1–50 days (Table 7).

**Table 7:** Length of hospital stays for patients with HF burns

Hospital stay (days)	Cases	
	N	Percent (%)
<10	148	73.63
10–19	23	11.44
20–29	12	5.97
30–39	14	6.97
40–49	3	1.49
>50	1	0.50

### Mortality

Two patients died of sudden cardiac arrest caused by severe fluoride poisoning. The overall mortality rate for HF burns in this study was approximately 1%.

## Discussion

The morbidity of HF burns varies among regions throughout the world [13]. In some areas, HF burns occur more frequently and have become one of the most common chemical injuries [8, 10]. Our study demonstrated an increased incidence of HF burns in western Zhejiang Province between 2004 and 2013, although a small surge was also observed in 2006 (Fig. 1). HF industries and derivatives have shown explosive growth in the past decade and play a vital role in the local economy. Consequently, the incidence of chemical burns, especially those caused by HF, has increased [10, 14, 15].

As observed in this study, the majority of chemical burns were work related, which was seen in the working-age population. This demographic was seen to be affected most frequently [7, 16, 17]. More than 95% of individuals with chemical burns were between the ages of 20 and 59 years (Table 1). Furthermore, most patients had low educational levels (primary and middle school education), and only a small number of patients had received tertiary education of college/university degrees or higher (Fig. 2). A low level of education may partly explain the higher incidence of HF burns in these patients. One case should be noted regarding two patients who were under the age of 10 years; and both of these children were victims of chemical burns in family workshops. In the past, family workshops, such as glass etching, were very popular in some regions of China. These establishments were usually contained within homes where workshops would be connected to the household private area, such as living room, allowing children easy access to dangerous chemicals and increasing the risk and likelihood of injury. In addition, more than 71% of chemical burns were work related, occurring primarily in workers in the fluoride industries, and stevedoring and transportation (Table 2). A recent study investigated acute HF exposure cases occurring in 1991–2010 using data collected from the Taiwan Poison Control Centre [18]. A total of 324 cases were identified, of which 80% were caused by occupational exposure, including those occurring in the semiconductor industry (61%), cleaning industry (15%), chemical and metal industries (13%), and other industries (11%). Some obvious differences in the occupational distribution of HF burns exist between existing data and those obtained within this study.

Workplace protection against chemical burns requires the use of personal protective equipment (PPE) and necessary professional skills and knowledge (received as training) when operating machines or handling dangerous chemicals. More than 95% of chemical burns assessed in this study occurred in the workplace and were caused by inadequate protection, machine problems, and inappropriate machine operation (Fig. 3). Thus, shortages in occupational education and training, machine maintenance, and production management exist and can partly explain the higher incidence of HF burns in western Zhejiang.

HF, in liquid or vapour form, has the potential to cause tissue corrosion and chemical poisoning. Six patients were injured due to hydrogen fluoride exposure, and all others were burned by aqueous HF solutions (Table 3). HF burns caused by highly concentrated acid were more common, followed by moderate and low concentrations of acid (Table 4). These characteristics may correlate directly with production techniques and transportation practises in various fluoride industries. In many enterprises, highly concentrated HF solutions are produced and used, and may also be delivered to various regions if required; these processes were seen to be major causes of HF burns due to the improper handling of chemicals, as previously explained. Furthermore, HF burns may be caused by exposure to mixed compounds (Table 3); such complex situations may affect physicians’ judgments and clinical decisions , leading to the incorrect management and treatment of chemical burns caused by exposure to HF [19].

Small burn areas were common in patients with HF burns (Table 5). The head and neck (37.36%) were the most common sites involved, followed by the hands (26.79%), legs (13.58%), and arms (10.57%). However, when burns involving the hands and arms were assessed together, the upper extremities (37.36%) were also the most common site of HF injury (Fig. 4), which parallels the results reported by Hatzifotis et al. [3] and Stuke et al. [17]. These authors assessed cases of patients with chemical burns, where most of the injuries involving the extremities could be prevented, once again emphasising the importance of correct occupational training, management and protection upon exposure to dangerous chemicals. In the study, by Hatzifotis et al. [3] and Stuke et al. [17], burns located on the head and neck were also usual; a result which also corresponded with the assessments of patients at our hospital department. Further analysis showed that burn injuries of this nature were caused by chemicals spilled or splashed from machines or pipes. As mentioned above, most HF burns occurred in the workplace and resulted from inadequate protection, machine problems, and inappropriate machine operation, which could explain the high frequency of HF burns affecting the head and neck. Accordingly, ocular burns were the most common accompanying injury.

In patients with fluoride poisoning, electrolyte imbalances, such as hypocalcaemia and hypomagnesaemia, occurred frequently, as fluoride ions bind to these metal ions to form insoluble salts in the body [13]. Hypocalcaemia and hypomagnesaemia can be rectified by calcium and magnesium supplementation in the clinic [20, 21]. Whether HF can cause hyperkalaemia is a matter of some controversy. Some experimental studies have shown that fluoride ions caused hyperkalaemia; when sodium fluoride was employed to investigate the effects of fluoride ions on potassium levels, hyperkalaemia was caused by inactivation of cellular sodium/potassium ATPase pumps and via the activation of sodium/calcium ion exchange [22]. However, different physiological mechanisms may be involved in patients with HF injuries. Some studies have reported that hypokalaemia occurs in patients with HF burns [22–25]. In our study, no such patient with hyperkalaemia was identified (Table 6), and this finding is paralleled in another recent epidemiological study conducted by Wu et al. [18] Hence, the exact mechanisms of HF on potassium metabolism remain unknown, and further studies are warranted. Inhibition of the ongoing absorption of fluoride ions is the key measure for the treatment of fluoride poisoning and its complications. Different methods have been developed to do so, including various decontamination methods using antidotes of calcium gluconate and others [13].

Seasonally, the distribution of HF burns remained similar in the summer, autumn, and winter; however, the number of burns decreased significantly in the spring compared with all other seasons (Fig. 5). A possible reason for this trend is the occurrence of many important holidays, such as the Chinese Spring Festival (during the spring season), which reduces the absolute working time in most fluoride enterprises.

The lengths of hospital stays and rates of surgical operation were related mainly to the severity of HF burns. Chemical burn severity is usually estimated by factors including HF concentration, exposure time, site of exposure, delay in therapy initiation, and amount of antidote delivered [2, 13, 26]. More than 90% of patients presented with small (<5% TBSA) burn areas, and most healed without surgical intervention, resulting in shorter hospital stays. Moreover, timely and correct pre-hospital treatment is also crucial to prevent the progressive deepening and poor prognostic outcome of burn wounds. Immediate water irrigation remains the recommended decontamination method for on-site treatment of chemical burns [27, 28] as not only does it aid the removal of residual chemicals from wounds, but it also reduces the risk of developing chemical poisoning. In addition to water irrigation, other decontamination methods, including rinsing with a calcium gluconate solution and using Hexafluorine^®^ solution have also been introduced. An increasing number of studies suggest that decontamination with Hexafluorine^®^ solution in a very early stage post-exposure (several seconds to minutes) works well [29]. After admission, an algorithm was established in our department to treat those patients with HF exposure, which has been well documented in another review [13]. The basic strategies for HF burns were paralleled to blocking the fluoride ions from infiltrating into deep tissues by emergent decontamination [13].

As this study is part of our epidemiological survey of chemical burns, its limitations have been clarified in previously published work [10]. Briefly, one limitation is the source of the data as all our data were collected from one hospital in one area, and only inpatients were included. Hence, the estimation of morbidity based on these data may contain errors. However, the data presented here remain valuable; as Quhua Hospital is the main medical centre for the treatment of chemical burns, and it receives the majority of patients with chemical burns in western Zhejiang Province. Additionally, the epidemiological characteristics described in this study have the potential to provide valuable information to encourage the upgrading of safety measurements and formulation of preventive strategies.

## Conclusions

To summarise; this epidemiological study presents characteristic findings related to HF burns in western Zhejiang Province, China. Firstly, the morbidity of HF burns in the past 10 years showed a gradual increase, which can be attributed to the development of local fluoride industries. Secondly, most HF injuries were work related and distributed in working-age patients. Thirdly, aqueous HF solutions, especially those of high concentration, were the most common chemical cause of HF burns. Furthermore, inappropriate operation of equipment, machine malfunctions, and inadequate protection were identified as the leading causes of HF burns in the workplace. The burn area was <5% of TBSA in more than 90% of patients, and the most common burn sites were the head, neck, and upper extremities. Approximately 17% of patients underwent surgical operation. Accompanying injuries should be detected and treated correctly in a timely manner. Lastly, electrolyte imbalances, such as hypocalcaemia, hypomagnesaemia, and hypokalaemia, occurred frequently in patients with HF exposure; however, hyperkalaemia was not encountered in this study. Based on these results, related enterprises and local authorities should be encouraged to upgrade their management policies and to provide the necessary occupational education and safety training to high-risk populations. The local government would benefit from the establishment of a long-term strategic plan to improve education and to enhance the management of hazardous chemicals. Moreover, strategies focusing on the production, transportation, and usage of HF should be enhanced further for labourers and professionals dealing with chemicals, including consideration of the details of injuries caused by HF and other mixtures. Early and correct pre-hospital treatment, such as water irrigation and application of antidotes should also be considered for the effective management and immediate treatment of HF burns. Thus, the education of workers to provide common emergency knowledge and skills is highly warranted.
